# Association of thyroid disease and intracranial meningiomas: a retrospective analysis with external validation

**DOI:** 10.3389/fonc.2025.1635097

**Published:** 2026-01-15

**Authors:** Jonathan B. Bell, Max A. Saint-Germain, Carlen A. Yuen, Andrew Wilmington, Laura Dresser, Matthew T. Walker, Christopher R. Trevino, Roberto Salvatori, Debraj Mukherjee, David O. Kamson, Ryan Merrell

**Affiliations:** 1 Preliminary Internal Medicine Program, University of Chicago (Endeavor), Evanston, IL, United States; 2 Department of Radiation Oncology, Sylvester Comprehensive Cancer Center, Miller School of Medicine, University of Miami, Miami, FL, United States; 3 Sidney Kimmel Comprehensive Cancer Center, Johns Hopkins University, School of Medicine, Baltimore, MD, United States; 4 Department of Neurology, University of Chicago, Chicago, IL, United States; 5 Divison of Neuro-oncology, Department of Neurology, University of California at Irvine, Irvine, CA, United States; 6 Division of Hematology, Oncology, and Cell Therapy, Rush University Medical Center, Chicago, IL, United States; 7 Department of Radiology, Endeavor Health, Evanston, IL, United States; 8 Department of Neurology, Endeavor Health, Evanston, IL, United States; 9 Division of Endocrinology and Pituitary Center, Johns Hopkins University, School of Medicine, Baltimore, MD, United States; 10 Johns Hopkins University, Department of Neurosurgery, Baltimore, MD, United States; 11 Department of Neurology, Johns Hopkins University, School of Medicine, Baltimore, MD, United States; 12 Department of Neurology, Vanderbilt University, Nashville, TN, United States

**Keywords:** meningioma, thyroid disorders, hypothyroidism, thyroid hormone replacement, neuro-oncology, thyroid nodular disease

## Abstract

**Background:**

The scope of comorbid conditions with meningiomas is understudied. There is limited published evidence to date suggesting higher prevalence of thyroid diseases in patients with meningiomas. This retrospective study was designed to evaluate this association.

**Methods:**

The medical records from 584 patients with intracranial meningiomas were reviewed. The prevalence of thyroid disease was calculated as well as odds ratios to compare patient cohorts. Subsequently, the results were externally validated using SlicerDicer data from a second institution’s electronic medical record.

**Results:**

Within the Endeavor Intracranial Meningioma cohort, thyroid disease was found in 196/584 (33.6%): hypothyroidism in 154/584 (26.4%), nodular disease in 34/584 (5.8%), thyroid cancer in 9/584 (1.5%), and hyperthyroidism in 8/584 (1.4%) patients. An additional 9/584 (1.5%) patients had a different thyroid disease or diagnosis. Our external validation with patients only from the second institution yielded significant association of meningioma with thyroid hormone medication use (OR = 6.1), hypothyroidism (OR = 7.5), thyroid cancer (OR = 14.6), hyperthyroidism (OR = 5.9), and thyroid nodules (OR = 10.8). Comparing meningioma patients to patients with glioblastoma yielded a similar association between meningioma and general thyroid disorders (OR = 2.1), thyroid hormone usage (OR = 2.1), hypothyroidism (OR = 1.7), thyroid cancer (OR = 3.1), and thyroid nodules (OR = 7.5). These observations were not confounded by female overrepresentation.

**Conclusions:**

This study confirms thyroid disease, particularly hypothyroidism, to be a common comorbid condition in patients with intracranial meningiomas in two independent datasets. Further research is needed to assess the role of thyroid disorders in the outcomes for patients with meningioma.

## Introduction

Meningiomas are the most common type of intracranial neoplasm, accounting for more than 37% of primary intracranial tumors, with an age adjusted incidence rate of 8.56 per 100,000 people (1). Meningiomas arise from the dural lining of the CNS and are graded from 1 to 3 according to the 2021 World Health Organization (WHO) classification system (2). Management of meningiomas includes observation, surgical resection, or radiation therapy; these treatments are effective for most tumors. Systemic therapy plays a limited role in treatment outside of clinical trials (3). Although most tumors are benign, some are malignant, and local invasion can be detrimental to patient quality of life, necessitating treatment (4). Patients who undergo successful surgeries for meningioma have worse 2-year employment rates compared to the general population and the role of comorbidities, such as depression have been implicated in these adverse outcomes (5).

Several genetic, environmental, and metabolic factors play a role in the development of meningiomas. Genetic syndromes such as neurofibromatosis type 2 (NF2), Li-Fraumeni syndrome, and Cowden syndrome are known to increase the risk of these tumors. Furthermore, exposure to radiation therapy significantly increases the risk of meningiomas (6). The diagnosis of meningioma has also been associated with hormonal changes, most notably estrogen and progesterone (7). Indeed, there is an ongoing debate regarding the possible association between hormone replacement therapy (HRT) and development of meningiomas with progesterone use being implicated in meningioma growth (8, 9). However, estrogen HRT has no clear evidence of affecting meningioma growth rates (10). In addition to hormonal changes, metabolic risk factors are being recognized as important drivers of tumor growth. A large retrospective study in an ethnically diverse population found that obesity, diabetes, and hypertension are all associated with meningiomas (11). More research is needed to identify other associations that could provide insight into modifiable risk factors for the development of these relatively common tumors.

Thyroid disease is a comorbid condition in certain patients with meningiomas. Retrospective studies from the US and the UK have previously found associations between meningiomas and thyroid disease (12, 13). Thyroid diseases, particularly hypo- and hyperthyroidism, are relatively common disorders affecting individuals in both iodine-deficient and iodine-replete countries. These conditions are associated with significant morbidity and mortality (14, 15). In the US, overt hypothyroidism (i.e. low T4) is found in 0.3-3.7% of the population, and hyperthyroidism has a prevalence of approximately 0.5% (16). Subclinical hypothyroidism (i.e., high TSH and normal T4) increases with age, is more prevalent in females, and does not necessarily require treatment (17). Thyroid function abnormalities are linked with metabolic dysfunction, another suspected driver of meningioma growth, through several complex molecular mechanisms (18). Thyroid dysfunction may be associated with insulin resistance and metabolic syndrome (19), and obesity has been linked to a higher incidence of meningiomas (20). Based on these observations, we sought to uncover whether there is an association between thyroid disease and intracranial meningiomas in a large dataset of adult patients from our institution. This study is one of the largest analyses of thyroid disease in patients with intracranial meningiomas in the United States.

## Materials and methods

The Endeavor Health (formerly NorthShore University HealthSystem) electronic medical record (EMR) entries from 2006 to 2012 were queried for ICD codes of intracranial meningioma. This study was approved by an appropriate institutional review board, and considering this only required retrospective chart review, informed consent was not required or obtained. A total of 584 patients with meningioma were identified after exclusion of alternative diagnoses (e.g., schwannoma). The electronic medical records were reviewed for each patient to identify diagnoses of thyroid disease including hypothyroidism, nodular disease (including multinodular goiters and solitary thyroid nodules), thyroid cancer, hyperthyroidism, and other thyroid disorders (such as thyroid cysts). Prevalence of thyroid disease was calculated in both males and females and were compared by constructing contingency tables to calculate odds ratios and using Fisher’s exact tests. GraphPad Prism (Version 8.4.3) and SPSS (Version 24.0) were used for statistical analysis. P values < 0.05 were considered statistically significant. The results were externally validated using SlicerDicer^®^ data from the Second Institution’s EMR system to compare meningioma patients to glioblastoma patients (brain tumor control) and patients who underwent thyroid testing between March 2018 and March 2023. An additional variable, thyroid hormone usage, was added in the external validation, as a proxy for clinically significant hypothyroidism, as not all patients diagnosed with hypothyroidism require hormone replacement therapy, such as in cases of subclinical hypothyroidism (21). Thyroid testing was determined by the presence of TSH lab components in SlicerDicer^®^. Odds ratios were adjusted for sex and age when comparing meningioma patients with patients who underwent thyroid testing. Age was dichotomized into 18–49 and ≥50 years old based on prior literature suggesting sharp rise in meningioma incidence past age 50 (1).

## Results

Patient characteristics are listed in **Table 1**. In the Endeavor Health System Meningioma Database, there was a high prevalence of thyroid diseases, with the most common being hypothyroidism, found in 154 (26.4%) patients. Nodular thyroid disease was also relatively common in 34 (5.8%) patients. Other thyroid disorders, including thyroid cancer and hyperthyroidism, were less common, each accounting for a small proportion of the thyroid disease burden.

**Table 1:** Patient characteristics (Endeavor Health System Meningioma Database).

Characteristics	Value (*%*)
Patients	584 (*100*)
Median age, years (range)	66 years (20-97)
Sex	
Male	150 (*25.7*)
Female	434 (*74.3*)
Meningioma	
WHO Grade 1	72 (*81.0*)
WHO Grade 2	16 (*18.0*)
WHO Grade 3	1 (*1.1*)
Multiple lesions	43 (*13.5*)
Prior RT (if available)	38 (*12*)
Thyroid disease	
Hypothyroidism	154 *(26)*
Nodular Disease	34 *(5.8)*
Thyroid cancer	9 (*1.5*)
Other	9 (*1.5*)
Hyperthyroidism	8 (*1.4*)

External validation of the Endeavor Health data analysis yielded similar findings. 749 patients with meningioma, 1,033 with glioblastoma, and 705,716 patients who received thyroid testing in the Second Institution’s general EMR population were found in SlicerDicer. When comparing the prevalence of thyroid disease between meningioma and glioblastoma patients, the odds ratios for meningioma patients were 2.1 (CI95%, 1.6-2.7) for thyroid hormone usage, 1.7 (CI95%, 1.3-2.2) for hypothyroidism, 3.1 (CI95%, 1.5-6.3) for thyroid cancer, and 7.5 (CI95%, 3.8-14.8) for nodular disease (**Table 2**; p ≤ 0.001 for all). In contrast, there was no significant difference in the prevalence of hyperthyroidism between meningioma and glioblastoma patients. Comparing thyroid disease in meningioma patients with that of the thyroid tested patient population of the Second Institution yielded an OR of 5.7 (CI95%, 4.8-6.9) for thyroid hormone medication use, 1.4 (CI95%, 1.1-1.7) for hypothyroidism, 3.3 (CI95%, 2.2-4.9) for thyroid cancer, 2.1 (CI95%, 1.3-3.4) for hyperthyroidism, and 4.0 (CI95%, 3.0-5.3) for thyroid nodule (**Table 3**; p<0.01 for all).

**Table 2:** Comparison of thyroid disease burden between meningioma and glioblastoma patients (external validation cohort).

Characteristics	Meningioma (%)	Glioblastoma (%)	Odds ratio (95% CI)	Fisher’s exact test
Thyroid disease	749	1033	–	*-*
Thyroid Medication Usage	153 (20.4)	114 (11.0)	2.1 (1.6-2.7)	*p < 0.001*
Hypothyroidism	125 (16.7)	112 (10.8)	1.7 (1.3-2.2)	*p < 0.001*
Nodular disease	51 (6.8)	10 (0.1)	7.5 (3.8-14.8)	*p < 0.001*
Thyroid cancer	24 (3.2)	11 (1.1)	3.1 (1.5-6.3)	*p = 0.001*
Hyperthyroidism	16 (2.1)	14 (1.4)	1.6 (0.8-3.3)	*p = 0.206*

**Table 3:** Comparison of thyroid disease burden between meningioma patients and the thyroid tested patient population in the external validation cohort.

Characteristics	Meningioma (%)	General patient population (%)	Odd ratio (95% CI)	Fisher’s exact test
Thyroid disease	749	705,716	–	*-*
Thyroid Medication Usage	153 (20.4)	30,221 (4.3)	5.7 (4.8-6.9)	***p < 0.0001***
Hypothyroidism	125 (16.7)	90,403 (12.8)	1.4 (1.1-1.7)	***p = 0.002***
Nodular disease	51 (6.8)	12,776 (1.8)	3.3 (2.2 to 4.9)	***p < 0.0001***
Thyroid cancer	24 (3.2)	7,106 (1.0)	2.1 (1.3 to 3.4)	***p = 0.01***
Hyperthyroidism	16 (2.1)	7,323 (1.0)	4.0 (3.0 to 5.3)	***p < 0.0001***

Bold values indicate statistical significance.

A subanalysis adjusting for age and sex when comparing meningioma patients to patients who received thyroid testing was performed. For female patients between the ages of 18 and 49, the significant odds ratios for female meningioma patients were 4.5 (CI95%, 1.9-10.4, p=0.003) for thyroid hormone usage and 11.0 (CI95%, 5.2 -23.0, p<0.0001) for nodular disease (**Table 4**). For females who were at least 50 years old, the statistically significant odds ratios for meningioma patients were 4.8 (CI95%, 3.9-5.9, p<0.0001) for thyroid hormone usage, 2.6 (CI95%, 1.6-4.3, p=0.0005) for thyroid cancer, 2.1 (CI95%, 1.2-3.6, p=0.02) for hyperthyroidism, and 3.7 (CI95%, 2.8-5.0, p<0.0001) for nodular disease (**Table 4**). Finally, for male patients between the ages of 18 and 49, the only statistically significant odds ratio for male meningioma patients was 10.4 (CI95%, 3.6-29.5, p=0.0009) for thyroid hormone usage, (**Table 5**). For males who were at least 50 years old, statistically significant odds ratios for meningioma patients were 4.8 (CI95%, 3.9-5.9, p<0.0001) for thyroid hormone usage, 3.4 (CI95%, 1.4-8.3, p=0.02) for thyroid cancer, and 3.4 (CI95%, 1.7-6.9, p=0.003) for nodular disease (**Table 5**).

**Table 4:** Age and sex adjusted subanalysis comparing thyroid disease burden between female meningioma patients and the female thyroid tested patient population in the external validation cohort.

Characteristics	Female meningioma (%)	Female patient population with thyroid testing (%)	Odd ratio (95% CI)	Fisher’s exact test
Thyroid disease			–	*-*
Thyroid hormone usage				
18–49 years old	6/68 (8.8)	3,775/178,267 (2.1))	4.5 (1.9 to 10.3)	***p = 0.003***
≥50 years old	116/464 (25.0)	16,413/252,941 (6.5)	4.8 (3.9 to 5.9)	***p < 0.0001***
Hypothyroidism				
18–49 years old	4/68 (5.9)	15,385/178,26 (8.6)	0.7 (0.2 to 1.8)	*p = 0.522*
≥50 years old	95/464 (20.5)	49,776/252,94 (19.7)	1.1 (0.8 to 1.3)	*p = 0.682*
Nodular disease				
18–49 years old	8/68 (11.8)	2,139/178,267 (1.2)	11.0 (5.2 to 23.0)	***p <0.0001***
≥50 years old	50/464 (10.8)	7,992/252,941 (3.2)	3.7 (2.8 to 5.0)	***p <0.0001***
Thyroid cancer				
18–49 years old	1/68 (1.5)	1,693/178,267 (0.9)	1.6 (0.2 to 11.2)	*p = 0.478*
≥50 years old	17/464 (3.7)	3,625/252,941 (1.4)	2.6 (1.6 to 4.3)	***p = 0.0005***
Hyperthyroidism				
18–49 years old	2/68 (2.9)	2,054/178,267 (1.2)	2.6 (0.6 to 10.6)	*p = 0.185*
≥50 years old	13/464 (2.8)	3,464/252,941 (1.4)	2.1 (1.2 to 3.6)	***p = 0.015***

Bold values indicate statistical significance.

**Table 5:** Age and sex adjusted subanalysis comparing thyroid disease burden between male meningioma patients and the male thyroid tested patient population in the external validation cohort.

Characteristics	Male meningioma (%)	Male patient population with thyroid testing (%)	Odd ratio (95% CI)	Fisher’s exact test
Thyroid disease			–	*-*
Thyroid hormone usage				
18–49 years old	4/33 (12.1)	992/75,446 (1.3)	10.4 (3.6 to 29.5)	***p = 0.0009***
≥50 years old	27/180 (15)	6,856/167,857 (4.1)	4.1 (2.8 to 6.2)	***p < 0.0001***
Hypothyroidism				
18–49 years old	4/33 (12.1)	3,601/75,446 (4.8)	2.8 (1.0 to 7.8)	*p = 0.071*
≥50 years old	22/180 (12.2)	19,900/167,857 (11.9)	1.0 (0.7 to 1.6)	*p = 0.818*
Nodular disease				
18–49 years old	1/33 (3.0)	338/75,446 (0.4)	6.9 (0.9 to 51.0)	*p = 0.138*
≥50 years old	8/180 (4.4)	2,259/167,857 (1.3)	3.4 (1.7 to 6.9)	***p = 0.003***
Thyroid cancer				
18–49 years old	1/33 (3.0)	365/75,446 (0.5)	6.4 (0.9 to 47.2)	*p = 0.148*
≥50 years old	5/180 (2.8)	1,404/167,857 (0.8)	3.4 (1.4 to 8.3)	***p = 0.018***
Hyperthyroidism				
18–49 years old	0/33 (0)	460/75,446 (0.6)	0	*p = 1*
≥50 years old	1/180 (0.6)	1,233/167,857 (0.7)	0.8 (0.1 to 5.4)	*p = 1*

Bold values indicate statistical significance.

Finally, an analysis of the prevalence of meningiomas in patients with thyroid disease versus thyroid tested patients without thyroid disease was performed. In the female patient population between the ages of 18 and 49 years, the significant meningioma odds ratios were 2.1 (CI95%, 1.6-2.7, p<0.0001) for females with hypothyroidism, 2.7 (CI95%, 1.9-3.9, p<0.0001) for nodular disease, and 2.3 (CI95%, 1.2-4.4, p=0.01) for thyroid cancer (**Table 6**). For females with thyroid disease aged 50 years or older, the significant meningioma odds ratios were 1.6 (CI95%, 1.4-1.6, p<0.0001) in females with hypothyroidism, 1.7 (CI95%, 1.6-1.9, p<0.0001) for nodular disease, 1.6 (CI95%, 1.3-2.0, p<0.0001) for thyroid cancer, and 0.5 (CI95%, 0.5-0.6, p<0.0001) for thyroid hormone usage (**Table 6**). For male patients between the ages of 18 and 49 years, the significant meningioma odds ratios were 4.5 (CI95%, 2.9-7.1, p<0.0001) for males with hypothyroidism, 4.1 (CI95%, 1.9-8.9, p<0.0001) for nodular disease, and 3.3 (CI95%, 2.1-5.2, p<0.0001) for thyroid hormone usage (**Table 7**). For males with thyroid disease aged 50 years or older, the significant meningioma odds ratios were 1.7 (CI95%, 1.5-2.0, p<0.0001) in males with hypothyroidism, 2.0 (CI95%, 1.6-2.5, p<0.0001) for nodular disease, 3.1 (CI95%, 2.2-4.4, p<0.0001) for thyroid cancer, and 0.6 (CI95%, 0.5-0.8, p<0.0001) for thyroid hormone usage (**Table 7**).

**Table 6:** Age and sex adjusted subanalysis comparing meningioma disease burden between female thyroid disease patients and the female thyroid tested patient population in the external validation cohort.

Characteristics	Female thyroid disease patients with meningioma (%)	Female, thyroid-tested, no thyroid disease (% with meningioma)	Odd ratio (95% CI)	Fisher’s exact test
Thyroid disease			–	*-*
Thyroid hormone usage				
18–49 years old	48/31,626 (0.2)	265/180,866 (0.1)	1.0 (0.8 to 1.4)	*p = 0.812*
≥50 years old	688/142,707 (0.5)	2,389/261,586 (0.9)	0.5 (0.5 to 0.6)	***p < 0.0001***
Hypothyroidism				
18–49 years old	69/23,956 (0.3)	231/165,670 (0.1)	2.1 (1.6 to 2.7)	***p < 0.0001***
≥50 years old	1,126/86,057 (1.3)	1,792/211,357 (0.8)	1.6 (1.4 to 1.7)	***p < 0.0001***
Nodular disease				
18–49 years old	33/8,590 (0.4)	248/175,538 (0.1)	2.7 (1.9 to 3.9)	***p < 0.0001***
≥50 years old	497/31,572 (1.6)	2,213/242,655 (0.9)	1.7 (1.6 to 1.9)	***p < 0.0001***
Thyroid cancer				
18–49 years old	10/2,890 (0.3)	265/179,209 (0.1)	2.3 (1.2 to 4.4)	***p = 0.013***
≥50 years old	101/6,469 (1.6)	2,477/257,906 (1)	1.6 (1.3 to 2.0)	***p < 0.0001***
Hyperthyroidism				
18–49 years old	3/2,688 (0.1)	267/178,752 (0.1)	0.8 (0.2 to 2.3)	*p = 0.8*
≥50 years old	54/4,642 (1.2)	2,507/258,080 (1)	1.2 (0.9 to 1.6)	*p = 0.2*

Bold values indicate statistical significance.

**Table 7:** Age and sex adjusted subanalysis comparing meningioma disease burden between male thyroid disease patients and the male thyroid tested patient population in the external validation cohort.

Characteristics	Male thyroid disease patients with meningioma (%)	Male, thyroid-tested, no thyroid disease (% with meningioma)	Odd ratio (95% CI)	Fisher’s exact test
Thyroid disease			–	*-*
Thyroid hormone usage				
18–49 years old	24/6,146 (0.4)	90/76,727 (0.1)	3.3 (2.1 to 5.2)	***p < 0.0001***
≥50 years old	131/47,865 (0.3)	731/172,977 (0.4)	0.7 (0.5 to 0.8)	***p < 0.0001***
Hypothyroidism				
18–49 years old	25/5,192 (0.4)	78/73,171 (0.1)	4.5 (2.9 to 7.1)	***p < 0.0001***
≥50 years old	216/31,836 (0.7)	605/152,954 (0.4)	1.7 (1.5 to 2.0)	***p < 0.0001***
Nodular disease				
18–49 years old	7/1,497 (0.5)	87/75,920 (0.1)	4.1 (1.9 to 8.9)	***p = 0.002***
≥50 years old	76/8,978 (0.8)	717/167,607 (0.4)	2.0 (1.6 to 2.5)	***p < 0.0001***
Thyroid cancer				
18–49 years old	2/669 (0.3)	93/76,373 (0.1)	2.5 (0.6 to 10)	*p = 0.2*
≥50 years old	33/2,477 (1.3)	748/171,556 (0.4)	3.1 (2.2 to 4.4)	***p < 0.0001***
Hyperthyroidism				
18–49 years old	1/594 (0.1)	93/76,274 (0.1)	1.4 (0.2 to 9.9)	*p = 0.5*
≥50 years old	2/1,620 (0.1)	767/171,733 (0.4)	0.3 (0.1 to 1.1)	*p = 0.06*

Bold values indicate statistical significance.

## Discussion

The etiology and pathogenesis of meningiomas and potential overlaps with thyroid dysfunction remain largely unknown (22). In this retrospective study with external validation, we show that thyroid disease may be associated with intracranial meningiomas, supporting previous research by others (11, 12). Within the Endeavor HealthSystem Intracranial Meningioma cohort, we also found that thyroid disease was more common in females than males, however this is expected given the previously established individual associations between the female sex, thyroid disease, and meningioma (**Supplementary Table 1**) (11, 15). After adjustment for age and sex in the External Validation cohort, meningioma patients did not have higher odds of having hypothyroidism; however, they were significantly more likely to be using thyroid hormone replacement therapy. This suggests that patients with meningiomas may be more likely to experience clinically significant hypothyroidism requiring treatment.

Thyroid hormones are necessary for normal brain development and cross the blood-brain barrier by binding to transmembrane transporters (23, 24). Mechanistically, it is unclear whether or how thyroid function might affect meningioma development or growth. However, hypothyroidism (the most commonly identified thyroid disorder in our cohort) is associated with metabolic dysfunction and possibly obesity, both risk factors for the development of meningiomas and other neoplasms (25). It is plausible that thyroid dysfunction may contribute to meningioma pathogenesis through adverse effects on metabolism, a mechanism previously proposed by Seliger et al., who found in their matched case-control retrospective analysis that both obesity and arterial hypertension, core features of metabolic syndrome, were associated with an increased risk of meningioma (26). These findings have been substantiated by numerous studies, which have consistently linked adiposity with elevated meningioma risk (7, 27–31).

Further potential mechanisms of thyroid dysfunction and meningioma risk may involve the interplay between obesity, elevated serum insulin (32) and insulin-like growth factor levels (33), and the excess production of estrogens from adipose tissue (34), all of which may contribute to meningioma risk, especially in women (35, 36). Chronic inflammation, impaired immune function, and increased oxidative stress, which are common in metabolic syndrome, may also play a role in meningioma tumorigenesis (37). Interestingly, while speculation exists that elevated insulin signaling may promote meningioma development, the literature remains mixed, as some studies have found positive, null, and negative associations between diabetes and meningioma risk (26, 38–40). Overall, while the existing literature on the topic of obesity, abnormal insulin signaling, and their association with increased meningioma risk may not be conclusive, our findings in conjunction with prior literature warrant further investigation into the mechanistic link between thyroid dysfunction, metabolic dysfunction, and meningioma development. A better understanding of these mechanisms may shed light on modifiable risk factors and inform future preventative or therapeutic strategies for this disease.

Considering that intracranial tumors are often treated with cranial irradiation, one possible confounding mechanism of thyroid dysfunction in meningioma patients is secondary to treatment, which has been previously suggested (41, 42). Our database did not specifically identify the diagnosis of central (secondary) hypothyroidism, and it is possible that a subset of hypothyroid patients had hypothyroidism due to pituitary dysfunction. Brain irradiation, especially, is a well-known cause of pituitary dysfunction (42). Alternatively, Schneider et al. suggested that patients can develop hypopituitarism as a result of neurosurgery alone (43). In this study, this complication occurred more frequently in patients who underwent neurosurgery compared to those who only received chemotherapy or radiotherapy, regardless of whether the tumor was centrally or non-centrally located (43). This finding suggests that brain trauma secondary to surgical treatment may play a pivotal role in the development of pituitary dysfunction (42). However, in this study, the higher prevalence of thyroid disorders in meningioma relative to glioblastoma is unlikely to be driven by interventional confounders. This is due to differences in standard-of-care between the populations: brain radiotherapy and chemotherapy are utilized nearly universally in glioblastoma but used in only a small fraction of meningiomas cases (44). It does remain possible that development of treatment-induced thyroid dysfunction is diagnosed at a lower rate in glioblastoma due to the shorter survival in this patient cohort. Additionally, these interventional mechanisms do not address the other thyroid issues possibly associated with meningiomas, such as hyperthyroidism, thyroid cancer, and thyroid nodules. Although strong individual associations exist between female sex, age, and both thyroid disorders and meningiomas (45), controlling for sex and age in our stratified analyses still uncovered higher odds ratios of thyroid disorders among meningioma patients relative to patients who underwent thyroid testing. This demonstrates that the observed relationship remains independent of sex and age as confounding variables. Interestingly, while this study mainly served to analyze the prevalence of thyroid disease in meningioma patients, similar associations were observed when analyzing the reverse, the prevalence of meningiomas in patients with thyroid disease, with significant associations found in all subgroups except for patients with hyperthyroidism, young males with thyroid cancer, and young females taking thyroid hormones. Of note, we observed a lower prevalence of meningiomas in both older male and female patients taking thyroid hormones, which may seem to contradict our earlier finding of increased thyroid hormone usage in patients with meningiomas when compared to the thyroid tested general population. However, it is important to note that these observations are fundamentally different, as the former examines the proportion of thyroid hormone users among meningioma patients, while the latter assesses the proportion of meningioma diagnoses among thyroid hormone users. These findings may reflect differing baseline risks, treatment behaviors, or ascertainment patterns with these subpopulations. Nevertheless, this study notes bidirectional associations between thyroid disease and meningiomas, and the unclear nature of these associations highlights the need for longitudinal analyses to assess causality and temporal relationships.

One potential source of concern in this study is detection bias, as meningioma patients may have been more likely to receive testing for thyroid function. However, our control population was limited to patients who received thyroid testing from the Second Institution, consequently minimizing this source of bias. Thus, overall, our findings suggest the potential existence of other unexplored mechanisms linking thyroid disorders to meningiomas that may be of clinical significance. Particularly, given the higher proportion of patients with thyroid hormone replacement (clinically significant hypothyroidism) in the meningioma cohort, thyroid screening could potentially be considered during the work up for meningioma, however, further research is required to clarify the nature of this association and determine whether formal thyroid screening guidelines for meningioma patients are justified. Additionally, the role of comorbidities, such as depression in adverse postoperative employment outcomes for meningioma patients has been described before (5), and thyroid dysfunction is potentially another, modifiable risk factor.

## Limitations

Although this study suggests an interesting link between thyroid disease and meningiomas, there are limitations that should be considered. Firstly, the study is retrospective in nature, making it difficult to establish a causal link between thyroid dysfunction and meningiomas. The study is also limited as the Endeavor Health analysis does not include a control group. However, our external validation of the results using the Second Institution’s EMR SlicerDicer data mitigates this limitation as it compares thyroid disease prevalence in meningioma patients and the thyroid tested patient population. The SlicerDicer data also strengthens the reproducibility of this study as it involves an independent cohort of patients, and the use of a brain tumor control cohort allows us to reduce the probability of confounding by intracranial interventions. Another limitation of this study is that the hypothyroidism diagnosis may include both primary and secondary hypothyroidism, which may be present in patients with meningiomas in the sellar region. Potential confounding variables include obesity. However, our data is in line with previous retrospective studies. Further research is needed to explore the potential role of thyroid function in meningioma pathogenesis, evaluating possible molecular drivers as well as effects on tumor volume and growth rate. Prospective studies tracking thyroid function over time may shed light on the importance of endocrine function on meningioma growth and development.

## Conclusions

Thyroid dysfunction is common in patients with intracranial meningiomas. Hypothyroidism is the most common thyroid disorder in this cohort. Although a mechanistic link has not been established, there is growing evidence suggesting an interplay between thyroid function and the development of meningiomas and other intracranial tumors. Future studies are needed to establish the impact of thyroid disease on meningioma outcomes.

## Data Availability

The original contributions presented in the study are included in the article/**Supplementary Material**. Further inquiries can be directed to the corresponding author.
