# Supplementary material for: Association of thyroid disease and intracranial meningiomas: a retrospective analysis with external validation
Source: Front Oncol. 2026 Jan 15;15:1635097. doi: 10.3389/fonc.2025.1635097 (PMC12851958; doi:10.3389/fonc.2025.1635097)
Supplement: Supplementary file 1 [file Table1.docx]

**Supplementary Table 1.** **Comparison of thyroid disease burden by sex in the Endeavor Health System Meningioma Database.**

| Characteristics | Female (%) | Male (%) | Odds Ratio (95% CI) | Fisher’s Exact Test |
| --- | --- | --- | --- | --- |
| Thyroid disease | 167 (38.5) | 29 (19.3) | 2.6 (1.7-4.0) | ***p < 0.0001*** |
| Hypothyroidism | 127 (29.3) | 27 (18.0) | 1.9 (1.2-3.0) | ***p = 0.0071*** |
| Nodular disease | 33 (7.6) | 1 (0.7) | 12.3 (2.1-126.5) | ***p = 0.0008*** |
| Thyroid cancer | 6 (1.4) | 3 (2.0) | 0.7 (0.2-2.5) | *p = 0.7007* |
| Hyperthyroidism | 8 (1.8) | 0 (0) | - | - |
